# Video-Assisted Thoracoscopic Lobectomy *Versus* Stereotactic Body Radiotherapy Treatment for Early-Stage Non-Small Cell Lung Cancer: A Propensity Score-Matching Analysis

**DOI:** 10.3389/fonc.2020.585709

**Published:** 2020-11-10

**Authors:** Baiqiang Dong, Xuan Zhu, Zekai Shu, Yongling Ji, Fangxiao Lu, Jin Wang, Ming Chen

**Affiliations:** ^1^ Department of Radiation Oncology, Cancer Hospital of the University of Chinese Academy of Sciences (Zhejiang Cancer Hospital), Institute of Cancer and Basic Medicine (ICBM), Chinese Academy of Sciences, Hangzhou, China; ^2^ Department of Radiation Oncology, Zhejiang Key Laboratory of Radiobiology, Hangzhou, China; ^3^ Department of Radiation Oncology, The First Affiliated Hospital, College of Medicine, Zhejiang University, Hangzhou, China; ^4^ Department of X-ray, Cancer Hospital of the University of Chinese Academy of Sciences (Zhejiang Cancer Hospital), Institute of Cancer and Basic Medicine (ICBM), Chinese Academy of Sciences, Hangzhou, China

**Keywords:** early-stage non-small cell lung cancer, video-assisted thoracoscopic surgery lobectomy, stereotactic body radiotherapy, propensity score matching, treatment outcome, adverse event

## Abstract

**Background:**

Compared the overall outcomes of video-assisted thoracoscopic surgery (VATS) *versus* stereotactic body radiotherapy (SBRT) for stage I-II non-small cell lung cancer (NSCLC).

**Methods:**

We retrospectively compared overall survival (OS), cancer-specific survival (CSS), locoregional control (LRC), and disease-free survival (DFS) at our institution between January 2012 and December 2016. Propensity score-matching was performed to reduce patient selection bias based on age, gender, Karnofsky performance score, Charlson comorbidity index, pulmonary function, and tumor diameter.

**Results:**

A total of 567 patients treated with SBRT (n = 109) or surgery (n = 458) were included. Of those, 104 patients were matched for further analyses. Median follow-up was 44 months. At 3 and 5 years, OS was 88.6 and 79.9% for SBRT, and 94.2 and 91.6% for surgery (p = 0.097). There were no differences noted in 5-year CSS (83.7 *vs.* 91.6%, respectively; p = 0.270). The cumulative incidence of LRC at 3 and 5 years was comparable (93.5 and 93.5% *vs.* 94.0 and 85.9%, respectively; p = 0.621). Differences in the rates of disease-free survival at 5 years were not statistically significant (79.0 and 80.5%, respectively; p = 0.624).

**Conclusions:**

This propensity score-matching analysis suggests that SBRT can be an alternative option to VATS lobectomy for stage I-II NSCLC.

## Introduction

The early-stage lung cancer is an increasingly diagnosed disease owing to the widespread use of low-dose computed tomography (CT) screening into routine care (1, 2). Lobectomy offers the best potential cure for operable patients with early-stage non-small cell lung cancer (NSCLC) (3, 4). However, numerous patients cannot withstand thoracotomy due to comorbidities or personal preference. The minimally invasive video-assisted thoracoscopic surgery (VATS) has been associated with lower complication and faster functional recovery compared with open lobectomy; hence, this approach has gained increasing attention in the previous decades (5–8). From a technological perspective, thoracoscopic lobectomy represents a paradigm shift in surgery. By reducing the surgery-related physiologic insult, minimally invasive surgery expands the pool of operable patients who were previously considered potentially inoperable (9).

Stereotactic body radiotherapy (SBRT), also termed stereotactic ablative radiotherapy, delivers high doses of radiation to restricted volumes over a limited number of fractions. Owing to the steep dose gradients, this approach allows for effective tumor ablation with preservation of the surrounding tissue (10). Both the National Comprehensive Cancer Network Clinical Practice Guidelines and European Society for Medical Oncology Consensus recommend SBRT as a non-surgical treatment option for stage I-II NSCLC (11, 12). Additionally, the introduction and continuous advancement of this approach promise to improve outcomes in potentially operable patients (13–17). Recently, in patients with stage I NSCLC, both the use of VATS and SBRT increased (9), therefore, it would be clinical important in decision making for early stage NSCLC patients, especially for those who might tolerate surgery, but at certain risk of surgery. However, no randomized trials comparing minimally invasive lobectomy *versus* SBRT have been completed by now and retrospective comparisons may be precluded by imbalances in baseline characteristics between both cohorts.

In this study, we performed a propensity score-matching (PSM) analysis to compare the outcomes of both treatments in patients with T1-2N0M0 NSCLC. Our results suggest that both approaches provide similar outcomes, which could provide some clarity to the appropriateness of SBRT and help design and support future randomized trials.

## Material and Methods

### Study Population

All patients undergoing SBRT or VATS lobectomy at Cancer Hospital of the University of Chinese Academy (Hangzhou, China) for T1-2N0M0 clinically confirmed lung cancer from 2012 to 2016 were evaluated. Clinically confirmed lung carcinoma was defined as a primary suspicious mass, part-solid, or ground-glass opacity nodule with speculated or smooth edges shown on CT images that persisted for ≥3 months and showed an increase in its longest axis. In patients in whom radiological results were equivocally correlated, endobronchial ultrasonography or mediastinoscopy was performed at the physician’s discretion. Additionally, all patients underwent staging bone scan and brain magnetic resonance imaging. A positron emission tomography/CT (PET/CT) was recommended for all patients and was deemed necessary for diagnosis when biopsy was not considered medically safe or the patient refused to undergo the procedure. All the radiology reviews were blinded to treatment type. Patients with a tumor diameter >5.0 cm or those with a biological effective dose (BED) <100 Gy were excluded. All disease staging was performed using the Union for International Cancer Control Tumor, Node, Metastasis system (7^th^ Edition). The indications were fully examined and discussed among thoracic surgeons and radiation oncologists, all multidisciplinary consultations were well documented.

### Treatment Procedures

Most patients with adequate pulmonary function (forced expiratory volume in 1 s and diffusing capacity greater than 35% predicted), arterial oxygen tension greater than 60 mmHg, arterial carbon dioxide tension less than 50 mmHg, and absence of other contraindicating medical comorbidities, according to the thoracic surgeon, were selected for VATS lobectomy. The operation was performed under general anesthesia with single-lung ventilation through a double lumen tracheobronchial tube on a lateral decubitus position, with a <5 cm access incision and full dissection and individual division of hilar structures. Mediastinal lymph node dissection was routinely performed for surgical staging. There were no conversions to open surgery.

Inoperable patients, according to the thoracic surgeon, and those who refused surgical resection were selected for SBRT. The whole course of SBRT was reported in our previous study (18–20). The gross tumor volume (GTV) included only the primary tumor; the internal target volume (ITV) was determined using CT with a four-dimension CT technique, and the tumor motion was assessed. The planning target volume (PTV) was defined as the ITV expanded by a 5-mm margin in each direction. The treatment plans were optimized to limit the administration of high doses to regions of organs at risk. The conformality and dose limits of normal tissues were set according to RTOG0236 (21). In the treatment, 80% iso-dose line was used as the prescribed dose to cover 95% PTV, and 100% iso-dose line was used to cover 100% ITV. Daily online cone beam CT-based volumetric image-guided radiotherapy using soft tissue target registration was applied prior to all SBRT sessions. The BED was calculated using BED*_α/β_*= nd (1+ *d/α/β*), where *n* = number of fractions, *d* = dose per fraction, and *α/β* = 10 Gy for the tumor.

### Data Collection

Demographic variables obtained from the electronic file database of Cancer Hospital of the University of Chinese Academy (Hangzhou, China) included age, gender, forced expiratory volume in 1 s to forced vital capacity ratio (FEV1/FVC%) and FEV1% predicted prior to treatment, Karnofsky Performance Status score (KPS), and Charlson comorbidity index (CCI). Tumor characteristics included diameter and histology. Complications in the surgery group and toxicity in the SBRT group were scored according to the Common Terminology Criteria for Adverse Events Version 4.0.

Post-treatment follow-up generally consisted of a CT scan of the thorax and upper abdomen performed within 2 months of treatment completion for the first and every 3 months for the first 2 years, and every 4–10 months thereafter. Primary tumor recurrence was diagnosed through histologic confirmation or enlargement of the local tumor on CT that continued for ≥6 months. A PET/CT was considered in case of high suspicion of recurrence. Notably, it is difficult to distinguish between pulmonary fibrosis and tumor recurrence, and intrapulmonary metastasis and secondary primary lung cancer. Therefore, a senior radiologist reviewed post-SBRT imaging to score patterns of failure. Local failure was defined as progression in the same lobe after SBRT or the bronchial stump or port site after surgery. Regional failure was defined as failure in ipsilateral hilar or mediastinal lymph nodes after either treatment. Distant failure indicated recurrence beyond locoregional failure.

The primary endpoint of the study was locoregional control (LRC) and cancer-specific survival (CSS). The latter was defined as the period from the date of treatment to the date of death due to lung cancer or treatment-related mortality. The analysis also focused on disease-free survival (DFS), overall survival (OS), and treatment-related toxicity. DFS was defined as the period from date of surgery or SBRT to the date of any failure, development of a new primary NSCLC, or date of death, with patients censored at the date of the last follow-up. OS was defined as the interval from the date of treatment to any death or the last follow-up.

### Statistical Analysis

The two-tailed *t* test was used for continuous variables. For non-normally distributed data, we used the Mann–Whitney *U* test for comparison. The *χ^2^* test was used for categorical variables. The Kaplan–Meier method was used to calculate the survival rates. PSM was performed using the R MatchIt package for Windows version. A two-tailed value of *p* < 0.05 denoted statistical significance.

## Results

### Patient Characteristics

**Figure 1:**
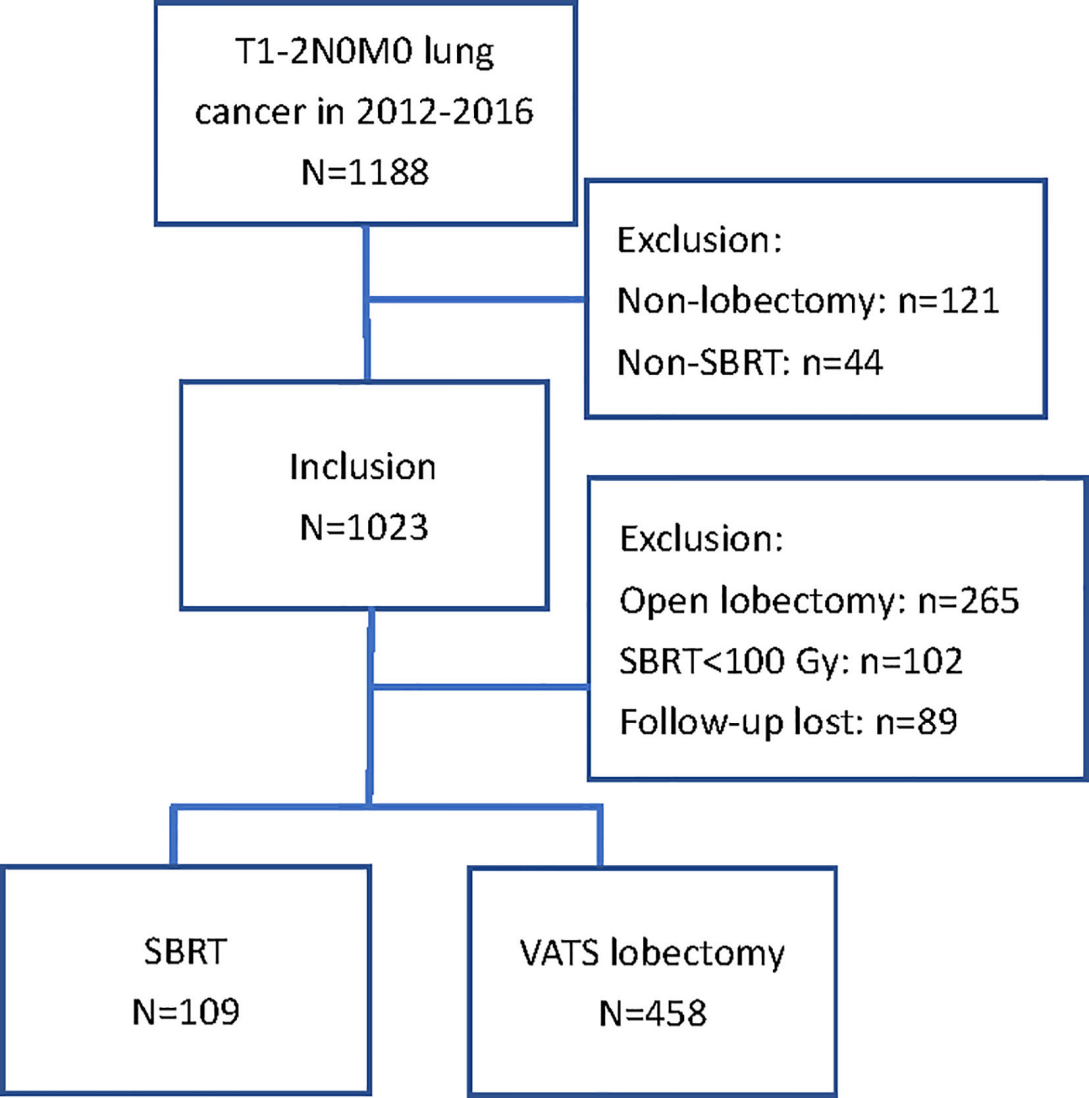
Description of study population; inclusions and exclusions.

A total of 567 patients treated with SBRT (n = 109) or surgery (n = 458) for stage I-II NSCLC were selected for matching. The exclusions and distribution between treatment methods in the included patients are shown in **Figure 1**. The baseline characteristics prior to PSM are summarized in **Table 1**. Patients who received SBRT exhibited significantly poorer respiratory function, higher CCI, and were older than those who underwent VATS lobectomy. Male patients preferred non-invasive therapy. Seventy-two SBRT patients (66%), including those without pathological confirmation, underwent PET/CT examination. The prescribed dose mainly delivered 50 Gy in five fractions (n = 74, 68%) or 50 Gy in four fractions (n = 24, 22%). Six patients (5%) received 60 Gy in eight fractions, four patients (4%) received 70 Gy in ten fractions, and one received 60 Gy in six fractions to the isocenter. All patients received a minimum BED*_10_* of 100 Gy (range: 100–120 Gy). In the surgery group, all had a complete (R0) resection with negative microscopic margins on final pathologic examination. The number of dissected lymph nodes was 13.3 ± 6.3 (mean±standard deviation), with 91% of patients having six or more nodes dissected.

**Table 1:** Characteristics of patients with early-stage NSCLC stratified according to treatment.

Variable	Overall cohort	SBRT	VATS lobectomy	*p*
	n (%)	n (%)	n (%)	
**Sociodemographics**				
Age (years)				**<0.001**
<65	320 (56)	19 (17)	301 (66)	
65–74	174 (31)	34 (31)	140 (30)	
≥75	73 (13)	56 (52)	17 (4)	
Gender				**<0.001**
Male	294 (52)	79 (73)	215 (47)	
Female	273 (48)	30 (27)	243 (53)	
FEV1% predicted				**<0.001**
<30	62 (10)	43 (39)	19 (4)	
30–49	101 (19)	24 (22)	77 (17)	
50–79	147 (26)	16 (15)	131 (29)	
≥80	257 (45)	26 (24)	231 (50)	
FEV1/FVC (%)				**<0.001**
≥70	547 (96)	91 (83)	456 (99)	
<70	20 (4)	18 (17)	2 (1)	
KPS				**0.033**
≥90	513 (90)	89 (82)	424 (92)	
<90	54 (10)	20 (18)	34 (8)	
CCI				**<0.001**
0	369 (65)	42 (39)	327 (71)	
1-2	170 (30)	58 (53)	112 (25)	
≥3	28 (5)	9 (8)	19 (4)	
**Tumor characteristics**				
Tumor size (cm)				0.201
≤2.0	328 (58)	56 (51)	272 (59)	
2.1–3.0	187 (33)	41 (38)	146 (32)	
3.1–5.0	52 (9)	12 (11)	40 (9)	

SBRT, stereotactic body radiotherapy; NOS, not otherwise specified; NSCLC, non–small cell lung cancer; Ade, adenocarcinoma; SCC, squamous cell carcinoma; FEV1, forced expiratory volume in 1 s; FEV1/FVC%, FEV1 and forced vital capacity ratio; CCI, Charlson comorbidity index; KPS, Karnofsky performance status.

In bold: the difference was statistically significant.

The matching process resulted in a final cohort of 104 patients (52 SBRT and 52 VATS patients) eligible for further analysis. The SBRT and VATS cohorts were similar in terms of age (median: 68 *vs.* 67 years, respectively), gender, tumor size (median: 2.0 *vs.* 2.0 cm, respectively), CCI, and respiratory function (**Table 2**).

**Table 2:** Characteristics of propensity score-matched patients.

Variable	SBRT	VATS lobectomy	*P*
	n (%)	n (%)	
Age (years)			0.455
Median (range)	68 (47–83)	67 (45–83)	
Gender			1.000
Male	31 (60)	31 (60)	
Female	21 (40)	21 (40)	
FEV1% predicted			0.471
Median (range)	79 (31–109)	80 (27–112)	
FEV1/FVC (%)			0.391
Median (range)	104 (31–125)	107 (54–119)	
KPS			0.918
Median (range)	90 (80–100)	90 (80–100)	
CCI			0.625
0	30 (58)	36 (69)	
1-2	10 (19)	6 (12)	
≥3	12 (23)	10 (19)	
Tumor size (cm)			0.411
Median (range)	2.0 (0.5–5.0)	2.0 (0.8–4.0)	

SBRT, stereotactic body radiotherapy; VATS, video-assisted thoracoscopic surgery; FEV1, forced expiratory volume in 1 s; FEV1/FVC%, FEV1 and forced vital capacity ratio; CCI, Charlson comorbidity index; KPS, Karnofsky performance status.

### Survival

**Figure 2:**
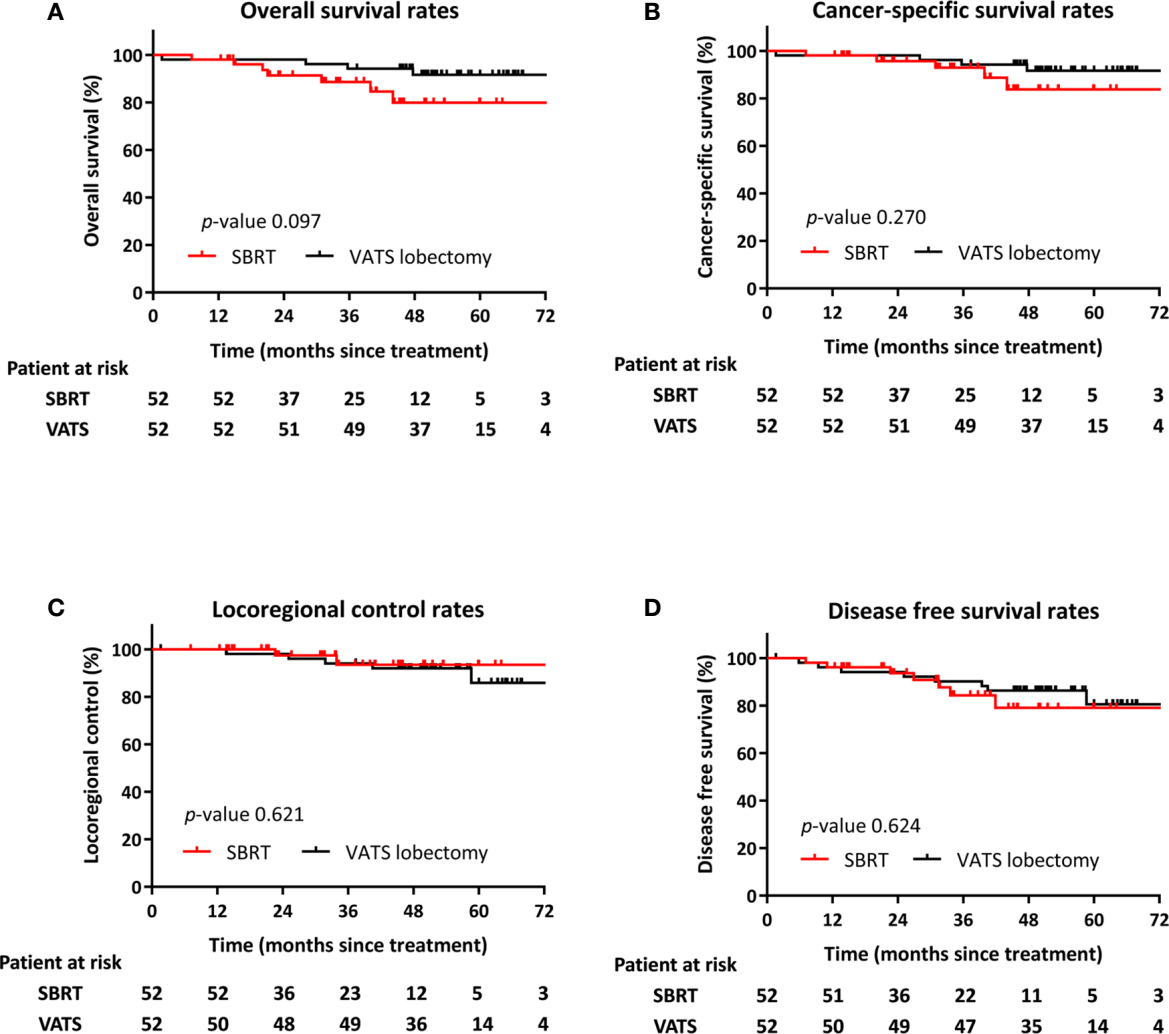
Kaplan–Meier curves following propensity score-matching for overall survival **(A)**, cancer-specific survival **(B)**, locoregional control **(C)**, and progression-free survival **(D)** of patients after VATS lobectomy or SBRT.

Follow-up data were complete until May 2019. The median follow-up was 44 months. Seven (14%), and four (8%) patients in the SBRT and VATS groups, respectively, died during the follow-up period. Death was tumor-related or attributed to other causes in two (18%) and nine (82%) patients, respectively. Other causes included one case of pneumonia and one death of unknown cause. OS was comparable between the two cohorts. The post-SBRT rates at 3 and 5 years were 88.6 and 79.9%, respectively, *versus* 94.2 and 91.6% for post-VATS (*p =* 0.097) (**Figure 2A**). There were no differences noted between SBRT and VATS for the 3- and 5-year CSS (92.9 and 83.7 *vs.* 94.2 and 91.6%, respectively; *p =* 0.270) (**Figure 2B**).

Locoregional failure occurred in five and two patients in the SBRT and surgery cohorts, respectively. The rates of LRC did not differ significantly between the groups (*p =* 0.621). The 3- and 5-year rates of LRC for radiotherapy and surgery were 93.5 and 93.5% *versus* 94.0 and 85.9%, respectively (**Figure 2C**).

Distal failures were observed in 13 patients, (i.e., six and seven patients in the SBRT and surgery groups, respectively). The majority of those (n = 9) were intrapulmonary metastases. The median time to any recurrence in patients treated with SBRT or VATS lobectomy was 24.9 and 27.9 months, respectively. DFS at 3 and 5 years was 90.2 and 80.5% for SBRT *versus* 79.0 and 84.3% for surgery (*p =* 0.624) (**Figure 2D**).

### Treatment Toxicity

**Table 3** outlines complications occurring after SBRT and VATS. Adverse events occurring within 6 weeks from SBRT were observed in eight patients (15%). The most commonly reported complication was increased dyspnea. One patient suffered grade 3 radiation pneumonitis. There was no grade 4–5 toxicity observed in the SBRT group. Systemic reactions were mainly fatigue, anorexia, and dyspnea during treatment; most of these resolved after symptomatic treatment. There was no mortality reported among SBRT patients in the 30-day period after treatment. In the surgery group, 29 patients suffered from complications (56%), and grade 1–2 complications were observed in 24 patients (46%). In the VATS group, the mortality rate during the 30-day period after resection was 2%, with one patient expiring due to multi-organ failure caused by septicemia (consequence of a severe lung infection). Five patients (10%) experienced grade ≥3 toxicity in the surgery group. One and two patients treated with SBRT and VATS lobectomy, respectively, required rehospitalization within 90 days.

**Table 3:** Complications after surgery and SBRT.

Toxicity/Complication	No. (%)	Grade 1	Grade 2	Grade 3
VATS lobectomy (n = 52)				
Pain	5 (10)	5	0	0
Cough	19 (37)	10	9	0
Shortness of breath	22 (42)	14	8	0
Hoarseness	3 (6)	3	0	0
Pneumonia	10 (19)	5	1	4
Pleural effusion	13 (25)	8	5	0
SBRT (n = 52)				
Radiation pneumonitis	18 (35)	14	3	1
Chest pain	3 (6)	3	0	0
Cough	26 (50)	19	7	0
Rib fracture	0 (0)	0	0	0
Shortness of breath	12 (23)	10	2	0
Hoarseness	2 (4)	2	0	0

SBRT, stereotactic body radiotherapy; VATS, video-assisted thoracoscopic surgery.

## Discussion

For decades, lobectomy has remained the accepted standard of care for early-stage NSCLC (22). However, this standard is currently being challenged by SBRT, especially for the treatment of elderly patients and those with clinically significant comorbidities. Earlier reports had shown that SBRT can achieve comparable outcomes to surgical resection even in operable patients (13, 16, 23, 24). Two prospective phase II clinical trials (JCOG 0403 and RTOG 0618), assessing SBRT in operable stage I NSCLC, showed that the OS at 3 years was 76–85%. This rate was similar to that reported for surgery (14, 15). However, most of these studies have been limited by different surgical approaches (thoracotomy and VATS), and mixed extents of resection (sublobar, lobectomy, bilobectomy, and pneumonectomy) (25). Fewer study focus on comparisons between SBRT and VATS (26). In addition, none of the four ongoing phase III trials comparing SBRT and surgical resection [VALOR (NCT02984761), POSTILV (NCT01753414), STABLE-MATES (NCT01622621), and RAXSIA (NCT03431415)] includes VATS. The relative effectiveness of treatments cannot be inferred in the absence of comparative data from modern minimally invasive techniques.

We performed a PSM pair analysis of outcomes of two potentially curative approaches for early-stage NSCLC. The PSM analysis identifies patients with similar characteristics, approximating the design of a randomized controlled trial. The results of this study indicated that the 3- and 5-year CSS rates associated with SBRT were comparable with those reported in patients who underwent VATS. Patients who received SBRT demonstrated promising LRC, despite undergoing only non-invasive staging of the lymph nodes. As expected, there were no significant differences observed between the two approaches in terms of LRC or DFS. The results reported in both groups were generally consistent with those currently available in the literature (27, 28). After 3 years, there appears to be a trend toward improved OS in patients who undergo surgery. We postulate that this finding was attributed to the poorer condition of patients who received SBRT *versus* that of patients who underwent VATS, despite the matching procedure. In addition, the long-term toxicity of SBRT should be monitored to assess whether non-tumor deaths are caused by therapeutic factors. We hope our findings will increase the impetus to expand the use of SBRT and to conduct high-quality clinical effectiveness trials.

It’s worth noting that our outcomes contradict to some of the recent suggestion that lobectomy is superior to SBRT. In some retrospective studies, patients selected for VATS have improved survival compared with SBRT (29–33). In these studies, routine systematic mediastinal lymph node dissection identified candidates for adjuvant chemotherapy, which may be associated with a significant difference in OS and CSS. In addition, the SBRT group consisted mainly of medically inoperable patients with poor pulmonary function and severe comorbidities, which accounts for their relatively low rates of survival. In D. Detillon’s study, the elderly patients with stage I NSCLC undergoing VATS lobectomy have a better OS than patients undergoing SBRT. However, due to the characters of the database, in their study, the cause of death was unknown. In addition, performance status and pulmonary function were not available in most of patients, which are very important for guiding treatment choice and prognosis (25). Further robust and long follow-up studies are warranted to demonstrate that SBRT may achieve comparable results with those reported after surgery.

There was no histopathologic proof of malignancy obtained in 22% of the patients who received radiotherapy. The probability of malignancy in 11 SBRT patients without a pretreatment pathological diagnosis was calculated using a combination of clinical, radiological, and PET/CT findings, as previously described. The national radiotherapy guidelines in the Netherlands indicate that patients without histologic confirmation undergo radiotherapy in case of: (a) a new or growing lesion shown on CT scans with characteristics of malignancy; (b) a high risk for developing lung cancer based on age and smoking history; and (c) PET/CT-positive lesions (17). The probability of benign disease in these patients is merely 4.3% (34). However, it remains our policy to obtain a pretreatment diagnosis in all patients, if possible.

An academic radiologist reviewed the post-SBRT imaging findings to score patterns of failure in this study. The majority of SBRT-induced lung injuries can result in a CT density change, which occasionally mimics tumor recurrence. This renders the distinction between recurrence and radiation fibrosis during follow-up challenging. In addition, it is difficult to differentiate between intrapulmonary metastases and secondary primary lung carcinoma. Therefore, it is important to identify and validate high-risk radiological features appearing on serial CT scans that suggest recurrence (i.e., enlarging opacity, continuous enlargement, enlargement after 12 months, bulging margin, linear margin disappearance, and loss of air bronchogram). The presence of high-risk radiological features coupled with a PET/CT maximum standardized uptake value >5 are highly suggestive of tumor recurrence (35, 36).

Toxicity is particularly important when considering options for the treatment of cancer with similar long-term survival. In this study, we observed limited toxicity in both groups. One patient developed a grade 3 complication (radioactive pneumonitis), and there were no deaths attributable to SBRT. In the surgery group, the incidence of supraventricular arrhythmias and empyema was lower, and grade 3 complications were observed in four patients. Of note, one patient (2%) expired due to perioperative infection within 30 days after surgery. The role of SBRT as a curative modality for early-stage NSCLC may become a more attractive option, considering the comparable overall clinical outcomes and low rates of treatment-related morbidity and mortality *versus* surgery.

A strength of our analysis is that the demographic- and tumor-matching factors at baseline were relatively comprehensive with limited variability. The strict 0.1 maximum caliper width for propensity score difference guaranteed an accurate PSM. Furthermore, the patient population truly reflected clinical practice, rather than being composed of selected, relatively suitable patients, which is often the case in clinical trials.

The limitations of this study should be acknowledged. Firstly, although the cohorts were accurately matched, this remains a retrospective study. Therefore, unidentified or unrecorded factors (e.g., histology and grade) may have played a role in selected patients. Squamous cell carcinoma and low-grade differentiation have been found associated with worse LRC and OS (37, 38). Secondly, patients receiving SBRT were clinically staged, while patients undergoing VATS were ultimately pathologically staged. Most SBRT patients did not undergo nodal staging or dissection in our analysis. Thus, it is likely that these patients were underestimated in this study, contributing to a pathological staging bias in favor of surgery. In addition, given the relatively small sample size and limited follow-up of our cohort, there is limited precision in estimating differences in outcomes across treatment groups. Finally, the optimal identification of acute and late treatment-related adverse events is another weakness of this study.

With the emergence of new technologies for the treatment of lung carcinoma (i.e., SBRT), and widespread introduction of lung cancer screening, thoracic surgeons will be faced with smaller lung lesions that may be amenable to these alternative treatments. The combination of these factors with the aging population characterized by more comorbidities indicates that thoracoscopic resection techniques present a favorable, more palatable option for many patients versus open procedures. Currently, there are insufficient data to assist clinicians and patients in reaching a decision regarding the optimal treatment for early-stage NSCLC. Retrospective reviews, may provide clues regarding the most appropriate management. The present findings provided useful information in answering these and other unresolved questions regarding SBRT.

## Conclusions

The results of this propensity score-matching analysis demonstrated that SBRT achieved comparable overall clinical outcomes to those observed with VATS. These data may provide a decision-making reference between healthcare providers and patients. Randomized trials investigating a number of minimally invasive techniques are required to accurately compare outcomes between these approaches.

## Data Availability Statement

The original contributions presented in the study are included in the article/supplementary material. Further inquiries can be directed to the corresponding authors.

## Ethics Statement

Ethical review and approval was not required for the study on human participants, in accordance with the local legislation and institutional requirements.

## Author Contributions

BD was responsible for project conceptualization, data collection and analysis, data interpretation, writing of the manuscript, and all manuscript revisions. XZ was responsible for project conceptualization, manuscript revisions, and editing of the manuscript. ZS was responsible for statistical analysis and editing of the manuscript. YJ was responsible for SBRT patient data collection and editing of the manuscript. FL was responsible for score the imaging results of follow-up patients. JW and MC were responsible for project conceptualization and editing of the manuscript and should be considered the guarantor for the article as a whole. All authors contributed to the article and approved the submitted version.

## Conflict of Interest

The authors declare that the research was conducted in the absence of any commercial or financial relationships that could be construed as a potential conflict of interest.
